# Qiime Artifact eXtractor (qax): A Fast and Versatile Tool to Interact with Qiime2 Archives

**DOI:** 10.3390/biotech10010005

**Published:** 2021-03-03

**Authors:** Andrea Telatin

**Affiliations:** Quadram Institute Bioscience, Gut Microbes and Health Program, Norwich Research Park, Norwich NR4 7UQ, UK; andrea.telatin@quadram.ac.uk

**Keywords:** bioinformatics, microbiome, metabarcoding, Qiime2, metadata

## Abstract

Qiime2 is one of the most popular software tools used for analysis of output from metabarcoding experiments (e.g., sequencing of 16S, 18S, or ITS amplicons). Qiime2 introduced a novel and innovative data exchange format: the ‘Qiime2 artifact’. Qiime2 artifacts are structured compressed archives containing a dataset and its associated metadata. Examples of datasets are FASTQ reads, representative sequences in FASTA format, a phylogenetic tree in Newick format, while examples of metadata are the command that generated the artifact, information on the execution environment, citations on the used software, and all the metadata of the artifacts used to produce it. While artifacts can improve the shareability and reproducibility of Qiime2 workflows, they are less easily integrated with general bioinformatics pipelines. Accessing metadata in the artifacts also requires full Qiime2 installation. Qiime Artifact eXtractor (*qax*) allows users to easily interface with Qiime2 artifacts from the command line, without needing the full Qiime2 environment installed (or activated).

## 1. Introduction

### 1.1. Bioinformatics Analysis of Metabarcoding Experiments

Metabarcoding is a widely adopted method to characterize the taxonomic composition and biodiversity of a microbial community [1]. The bioinformatic analysis of amplicon sequences generated by metabarcoding experiments is a complex workflow involving several steps (from the initial trimming the *locus*-specific primers from the raw reads to the generation of a contingency table). One of the most-used packages for the analysis of amplicon sequences is Qiime2 (Quantitative Insights into Microbial Ecology) [2], an extensible platform that includes all the tools required for the analysis of raw reads in a coherent and highly structured environment. Qiime2 provides the framework to integrate different tools (e.g., *cutadapt* [3] to remove the primers, and *DADA2* [4] to identify the representative sequences) in a coherent workflow, sometimes providing alternative tools to perform the same task (e.g., *deblur* [5] as an alternative to DADA2).

### 1.2. The Artifact Format Introduced by Qiime2

*Qiime2* introduced a new data format that the authors named ‘artifact’, consisting of a compressed archive containing structured metadata and labelled with a unique identifier. Each artifact has two attributes to define the format of the data files it carries: *type* and *data format*. These two attributes ensure that the appropriate input artifact is fed to each program or plug-in. For example, the plug-in to assign taxonomy to representative sequences using BLAST [6] (called *classify-consensus-blast*) will require as input an artifact for the query sequences and taxonomical database, both in *FeatureData[Sequence]* format, and a set of taxonomy labels in the *FeatureData[Taxonomy]* format. Each program or plug-in in Qiime2 requires input files to be packaged in an artifact of the appropriate type, and they will produce as output one or more artifacts.

All artifacts keep track of all their ‘ancestors’ (artifacts and tools used to generate it) and store the bibliography to cite for all the tools used.

The metadata stored in Qiime2 artifacts, and their integration within the Qiime2 framework, make this file format an important step toward making bioinformatics analyses shareable and reproducible [7].

However, only Qiime2 currently makes use of this archive file format, and the APIs to parse its metadata are only bundled with the whole package. This makes it complicated to extract or retrieve metadata for collaborators that not relying on this software for their analyses (for example, to perform secondary or downstream analysis starting from Qiime2 output artifacts). This limitation becomes increasingly apparent because Qiime2 releases are frequent and artifacts produced with a version are not necessarily compatible with artifacts produced by another version. In addition to this, there are no Qiime2 tools to check metadata from multiple artifacts. These shortfalls are hindering the exploitation of the full potential of artifacts for optimal reproducibility and integration of bioinformatics workflows.

### 1.3. Extending the Usability of Artifacts Beyond the Qiime Suite

Qiime artifacts currently live inside a Qiime workflow as they are tightly bound to the Qiime suite. However, there are two scenarios where the adoption of the artifact format outside the canonical Qiime2 workflow could be of interest.

A first use case could be the necessity (or the interest) to use artifacts produced by a Qiime2 pipeline to perform further analyses, or at least the possibility to explore their content, even when the software is not installed. Qiime2 provides a tool to extract files from artifacts, but it is limited to handling a single artifact at the time and exposes to the user only a fraction of the available information.

A second, more ambitious, scenario would be the adoption of the artifact format for analyses unrelated to metabarcoding. This possibility poses several challenges (for example, the discussion over what new formats would be required and how to validate them) and yet could bring the benefits of artifacts to new fields. In my opinion, microbial isolate genomics could be the first field trying to adopt this idea for the higher chance of researchers in the field of being familiar with Qiime2 to the relatively small size of most files if compared—for example—with human genomics.

To explore these possibilities, a standalone and fast tool to work with Qiime2 artifacts has been developed and released under the GPL-3.0 license, offering several ways to read metadata and extract the data from existing Qiime2 artifacts, and first implementing the possibility of generating new artifacts unrelated to microbiome studies (specifically, HTML reports).

## 2. Methods

*Qax* is a command-line program written in the *Nim* programming language (https://nim-lang.org, accessed on 26 February 2021) [8], a compiled language that allows rapid development of fast programs and is proving itself of particular interest for development of bioinformatics programs [9,10]. *Qax* has a modular architecture with a core parsing unit based on the standard ‘zip/zipfiles’ module, and shared across the subprograms of the suite, namely: *list*, *extract*, *cite*, *provenance* (to extract information or data from existing artifacts) and *make* (to generate Qiime2 visualizations starting from a static HTML website). The program only depends on the widely available ‘libzip’ library.

Pre-compiled, static binaries for Linux and macOS are available for download from the GitHub repository (https://github.com/telatin/qax, accessed on 26 February 2021), and the program can be easily installed from the BioConda repository [11].

The documentation, with usage examples, is available via GitHub pages at https://telatin.github.io/qax, (accessed on 26 February 2021).

## 3. Results

The *qax* program is comprised of a set of six subprograms: *list*, *extract*, *cite*, *provenance, view*, and *make.*

### 3.1. Listing Artifacts and Their Properties

**Figure 1:**
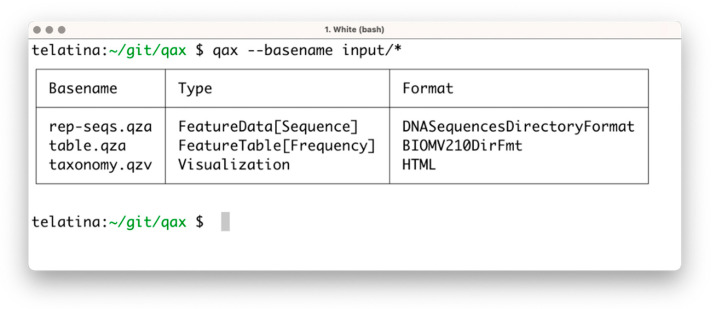
Screenshot of the execution of ‘qax list’ with multiple artifacts. If needed, more information can be printed using the ‘--all’ command argument.

The *list* subprogram parses a set of artifacts and returns a tabular summary of the properties of each artifact, acting as an ‘artifact-specific’ replacement of the *ls* command. In contrast to the ‘qiime tools peek’ command, provided by Qiime2, *list* can process multiple artifacts at once, and is more than 100 times faster. This utility can be used to prepare a tabular view of a list of artifacts and their attributes (including the unique identifier, called UUID, data format, and artifact type). An example of the execution is shown in Figure 1.

### 3.2. Extracting Data Files from Artifacts

Each artifact contains a bioinformatic dataset in a standard format that has the potential to be further analyzed outside Qiime. The *extract* command, as the name implies, provides fast access to the datasets and a convenient way to extract multiple artifacts at once. Qiime2 produces two different types of artifacts: regular artifacts (with the. *qza* extension) and visualization artifacts (with the. *qzv* extension), the latter being static HTML pages with a visual summary of a regular artifact. The *extract* module creates new directories for each artifact, which itself is composed of multiple files (i.e., all the visualization HTML files and some artifacts with multiple files, for example, the input FASTQ reads), whereas single files of interest will be extracted directly.

If the artifacts only contain a single data file (as is the case with feature tables and representative sequences, to name the most common examples), it will be extracted inheriting the name from the artifact filename, rather than preserving the standard name. This enables extraction of multiple artifacts at once, and organization of the output files in a single output directory.

**Figure 2:**
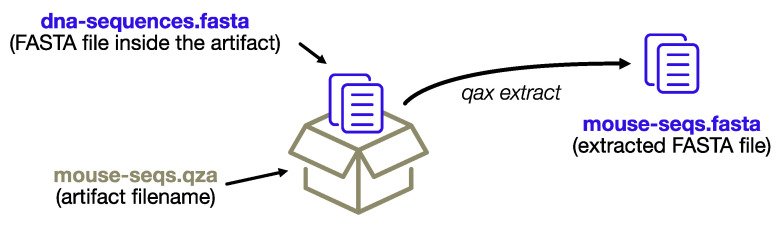
The extract subprogram will extract data files without re-creating the complex directory structure of the artifact format, conveniently renaming the files using the name of the artifact archive itself.

The process is depicted in Figure 2.

### 3.3. Extracting Bibliography from Artifacts

Each Qiime2 module provides citations for the software and resources that it uses and stores the citations in BibTeX format inside the artifacts. The *cite* module allows extraction of all citations from a list of artifacts removing duplicate citations, thus effectively enabling preparation of the bibliography for complete and reproducible Qiime2 analysis.

This module can be a useful addition to automatic workflows to generate the bibliography at the end of the execution.

### 3.4. Display and Plot Artifacts Provenance

Each artifact stores the metadata of all the artifacts used to generate it, back to the initial ‘importing’ steps. The only method provided by Qiime2 to access this ancestry is via the web browser using the convenient JavaScript viewer hosted at https://view.qiime2.org, (accessed on 26 February 2021). This limits programmatic access to the provenance trail. The *provenance* subprogram will print the list of artifacts used to generate the current one being inspected.

**Figure 3:**
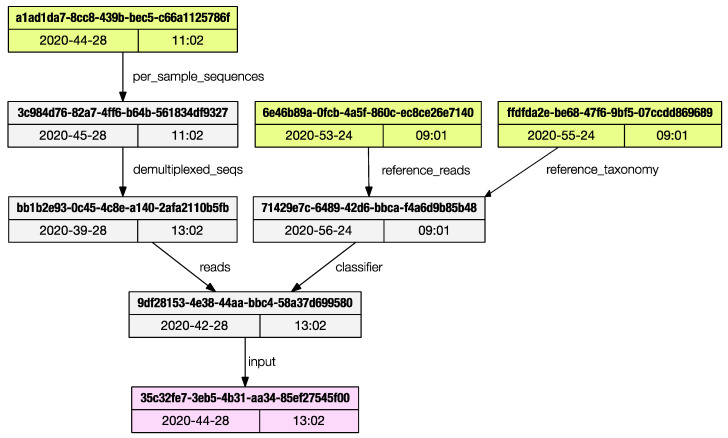
The *provenance* subprogram can render a publication grade provenance graph in PDF format.

The regular output is a list of artifacts UUIDs (universally unique identifiers) and their properties are printed to the terminal, and the user can save the graph in dot language [12]; if the ‘dot’ program is installed in the system, a publication grade PDF (as in Figure 3) can be directly generated.

### 3.5. Display the Content of an Artifact

Some artifacts contain one or more text files that can be easily inspected from the command line. The subprogram *view* allows the user to stream the content of one (or more) text files from artifact data.

### 3.6. Generate a Visualization Artifact to Distribute HTML Reports

The main use of *qax* is to interact with artifacts generated by Qiime2, except for *make* that will convert a directory containing an HTML document into a Qiime2 visualization artifact. This module enables an easier distribution of multi-file reports (pages, images, scripts and stylesheets) in a single compressed file.

This is the first tool that adopts the archive structure of Qiime2 artifacts enabling its use for bioinformatics workflow that do not involve Qiime2. The nature of visualization artifacts allows its use in any bioinformatics pipeline where a report is generated.

## 4. Discussion

The *qax* tool is a fast way to extract metadata from Qiime2 artifacts without installation (or activation) of the whole Qiime2 framework. This enables access to the artifacts by bioinformaticians using alternative toolkits and provides a convenient tool for complex pipeline development.

*Qax* can improve the user experience when working from the command line with Qiime2 artifacts, when performing amplicon analysis combining Qiime2 and other tools [13], and it is a convenient tool that can be used for automatic pipeline development (to generate the bibliography, to summarize metadata for all the generated artifacts, or to extract the data from artifacts for easier downstream analysis with third party tools) enhancing traceability and reproducibility of the analyses.

## 5. Conclusions

The *qax* tool is a fast, lightweight, and easy to install utility to interact with Qiime2 artifacts, allowing their use in downstream analyses with third party packages. The artifact format has the potential of being used and adopted outside the Qiime2 community in all those cases where traceability and reproducibility are important elements.

## Data Availability

The data and software presented in this study are openly available in Zenodo, at https://doi.org/10.5281/zenodo.4564269, accessed on 26 February 2021.
